# Analytical method for the determination of organic acids in dilute acid pretreated biomass hydrolysate by liquid chromatography-time-of-flight mass spectrometry

**DOI:** 10.1186/s13068-014-0145-3

**Published:** 2014-10-08

**Authors:** Ana B Ibáñez, Stefan Bauer

**Affiliations:** Energy Biosciences Institute, University of California, Berkeley, CA 94720 USA

**Keywords:** Organic acids, Dilute acid pretreatment, Inhibitors, Liquid chromatography, Mass spectrometry, Ion exchange chromatography, Biomass

## Abstract

**Background:**

For the development of lignocellulosic biofuels a common strategy to release hemicellulosic sugars and enhance the enzymatic digestibility of cellulose is the heat pretreatment of biomass with dilute acid. During this process, fermentation inhibitors such as 5-hydroxymethylfurfural, furfural, phenolics, and organic acids are formed and released into the so-called hydrolysate. The phenolic inhibitors have been studied fairly extensively, but fewer studies have focused on the analysis of the organic acids profile. For this purpose, a simple and fast liquid chromatography/mass spectrometry (LC/MS) method for the analysis of organic acids in the hydrolysate has been developed using an ion exchange column based on a polystyrene-divinylbenzene polymer frequently used in biofuel research. The application of the LC/MS method to a hydrolysate from *Miscanthus* has been evaluated.

**Results:**

The presented LC/MS method involving only simple sample preparation (filtration and dilution) and external calibration for the analysis of 24 organic acids present in dilute acid pretreated biomass hydrolysate is fast (12 min) and reasonably sensitive despite the small injection volume of 2 μL used. The lower limit of quantification ranged from 0.2 μg/mL to 2.9 μg/mL and the limit of detection from 0.03 μg/mL to 0.7 μg/mL. Analyte recoveries obtained from a spiked hydrolysate were in the range of 70 to 130% of the theoretical yield, except for glyoxylic acid, malic acid, and malonic acid, which showed a higher response due to signal enhancement. Relative standard deviations for the organic acids ranged from 0.4 to 9.2% (average 3.6%) for the intra-day experiment and from 2.1 to 22.8% (average 8.9%) for the inter-day (three-day) experiment.

**Conclusion:**

We have shown that the analysis of the profile of 24 organic acids present in biomass hydrolysate can be achieved by a simple LC/MS method applying external calibration and minimal sample preparation. The organic acids eluted within only 12 min by isocratic elution, enabling high sample throughput. Repeatability (precision and accuracy) and recovery were sufficiently accurate for most of the organic acids tested, making the method suitable for their fast determination in hydrolysate. We envision that this method can be further expanded to a larger number of organic acids, including phenolic acids such as *p*-coumaric acid and ferulic acid and other molecules depending on the researchers’ needs.

## Background

In the quest for renewable and sustainable energy, lignocellulosic biomass, such as herbaceous plants and hardwoods and softwoods, has been shown to be a promising feedstock for the production of second generation biofuels [1]. Lignocellulosic biomass essentially consists of the polysaccharides cellulose and hemicellulose and the aromatic macromolecule lignin. These compounds are present in the plant cell wall as a three-dimensional network giving the plant structure, stability, and resistance.

Pretreatment of the biomass is necessary in order to overcome this recalcitrance and facilitate degradation of polymeric structures [2-4]. In particular, the pretreatment methods aim to improve the conversion efficiency of the plant cell wall polysaccharides into fermentable monosaccharides by reducing the cellulose crystallinity or by simply splitting the carbohydrates and lignin for separate downstream processing technologies.

Various pretreatment methods have been developed for this purpose, comprising alkaline, acidic, or oxidative conditions (for a review see [2-4]). Dilute acid pretreatment is the most common pretreatment method and results in an almost complete solubilization of hemicellulose and a high enzymatic digestibility of the cellulose in the pretreated biomass. The acidic conditions and the higher temperature applied during this process also lead to degradation of the released monosaccharides and the lignin polymer [5]. These degradation products comprise compounds such as phenolics, furans, and organic acids which are inhibitory to fermenting microorganisms [6,7].

Whereas the phenolic inhibitors have been studied fairly extensively (see, for example, [8,9]), fewer studies have focused on the analysis of organic acids present in hydrolysate [10-13]. The predominant organic acids found in the hydrolysate after dilute acid (and other) pretreatment are acetic acid (released from acetate groups of hemicellulose and lignin) and levulinic and formic acid (both mainly derived from sugar degradation) [6,7]. Besides these, other organic acids are also observed, although in lower concentrations [10,11]. However, these compounds add up to the overall organic acid loading and can even contribute to synergistic toxic effects. A variety of analytical techniques have been developed for the measurement of organic acids, predominantly involving chromatography and capillary electrophoresis [14,15]. Although gas chromatography methods exist [14,16], liquid chromatography (LC) is the preferred technique, since it does not require derivatization. Many different stationary phases have been tested for this purpose, including reversed-phase [10,12,13,17-21], normal phase [22-24], and ion exchange [25-36]. If available, liquid chromatography coupled to a mass spectrometer results in specific detection of individual organic acids and unambiguous compound confirmation in contrast to refractive index (RI), ultraviolet (UV), or electrochemical detection. This is especially advantageous when the analysis has to be performed on samples with a complex matrix including potentially interfering compounds such as those found in hydrolysates. Only a few studies exist that apply mass spectrometry (MS) and also cover methodical approaches including basic method validation steps for the analysis of organic acids in pretreatment hydrolysates [12,13,19]. Chen *et al.* used reversed-phase chromatography and UV detection for the analysis of both aliphatic and phenolic acids and aldehydes after an organic solvent (methyl tertiary butyl ether) extraction step [12]. The method was further revised by a combination of UV and triple quadrupole MS detection to improve the specificity of the analysis [19]. This method was applied by Du *et al.* [10] for the measurement of both aliphatic and phenolic acids and aldehydes after a variety of pretreatments and also by Chundawat *et al.* [11] for the analysis of decomposition products formed by ammonia fiber expansion and dilute acid pretreatments. A single quadrupole MS method for formic acid and acetic acid was reported by Davies *et al.* [13].

One of the most popular types of liquid chromatography column used in biomass conversion research is a polymer-based matrix of polystyrene-divinylbenzene (for example, BioRad Aminex® HPX-87H, Phenomenex Rezex™-RFQ) [28,37]. This type of column provides good separation of simple sugars (such as glucose and xylose), many organic acids, alcohols (for example, ethanol and *n*-butanol), and sugar degradation products (such as 5-hydroxymethylfurfural and furfural). It only requires acidified water as the mobile phase, has excellent pH stability, and requires minimal sample preparation. For mass spectrometry coupling, the commonly used sulfuric acid is replaced with the volatile formic or acetic acid [34,38]. With this setup, organic acids have been analyzed [33-35,38,39], but its application to the analysis of organic acids in hydrolysate has had only very limited study [13]. We therefore evaluated the applicability of measuring organic acids in hydrolysate without any extraction or derivatization steps or the use of internal standards by applying a simple isocratic elution and time-of-flight mass spectrometry detection for high mass-accuracy compound confirmation.

## Results and discussion

**Figure 1:**
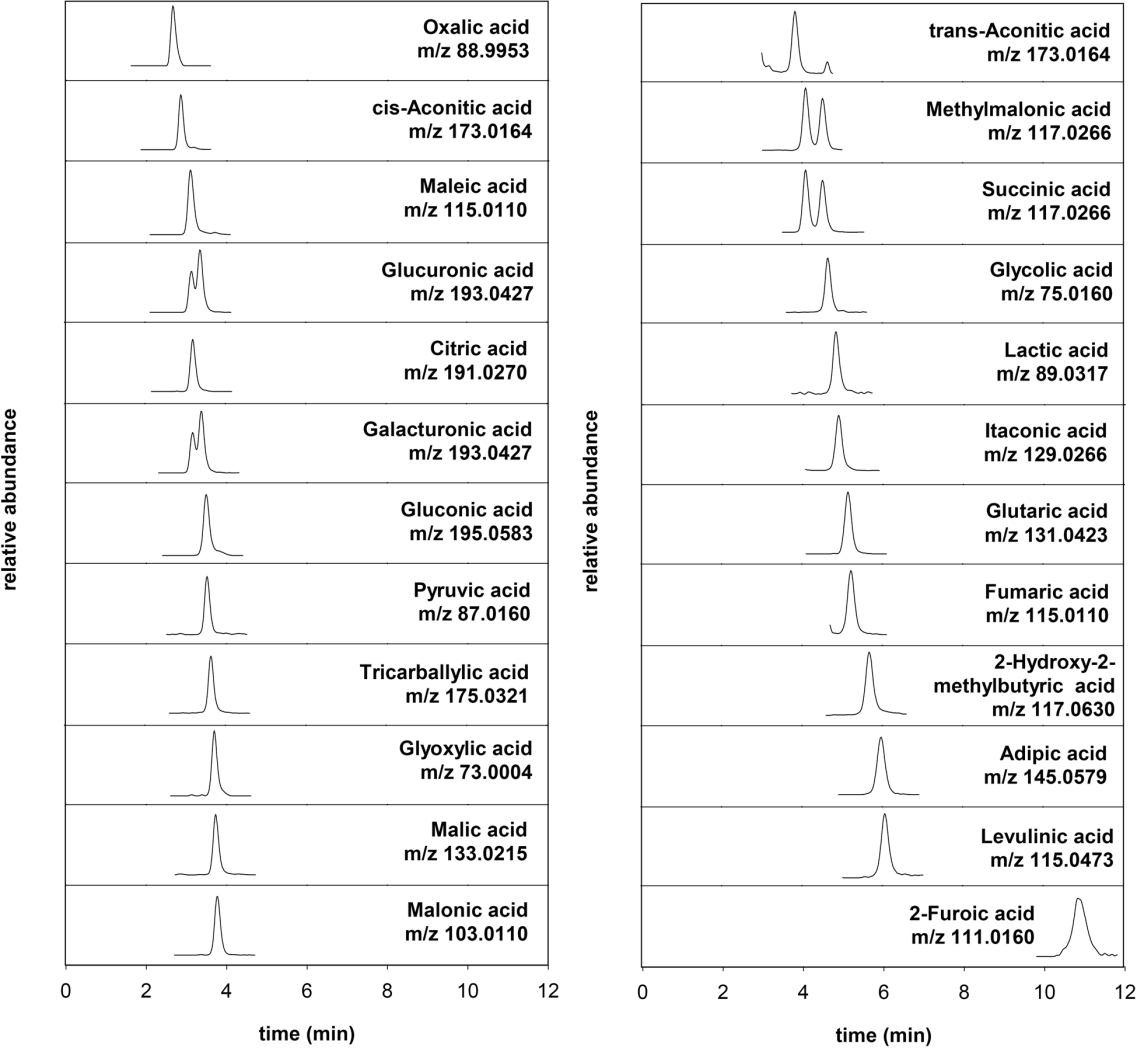
**Extracted negative ion chromatograms for the deprotonated organic acids [M - H]** ^**-**^ **based on the theoretical mass-to-charge ratio used for detection and quantification.** A standard mixture comprising all 24 organic acids was used. Therefore, extracted ion chromatograms show double peaks for the isobaric pair glucuronic/galacturonic acid and methylmalonic/succinic acid. For these pairs, glucuronic acid and methylmalonic acid eluted before their isobaric counterpart, respectively.

A commercially available ion exclusion column packed with a cation exchange resin based on a polystyrene-divinylbenzene polymer was used for the analysis. This column type has excellent stability at acidic pH and, in our experience, results in very reproducible retention times which are almost unaffected by other matrix components. Since the widely employed non-volatile eluent modifier sulfuric acid is not compatible with mass spectrometry detection, the volatile formic acid was used at a concentration of 0.5% (v/v) [34]. Acetic acid was also used in other works without any advantages in sensitivity in one study [34], although with enhanced sensitivity in another [38]. Lowering the formic acid concentration did not significantly change the retention times, but it can lead to a lower background signal and higher sensitivity [34]. The 0.5% formic acid was kept to ensure appropriate acidity in order to keep the organic acids in their non-ionized state for chromatography. The flow rate of 0.3 mL/min was chosen based on a reasonable compromise of sensitivity and time. A lower flow rate did not result in a better chromatographic separation of the analytes, but it extended the analysis time (data not shown). The mass spectrometer source parameters were varied in the range of 285 to 385°C for the source temperature, 75 to 175 V for the fragmentor voltage, and 3,000 to 4,000 V for the capillary voltage. Despite the aqueous mobile phase, a lower source temperature (285°C), combined with a low fragmentor (75 V) and capillary (3,000 V) voltage, was the best compromise for the detection of the organic acids under study. These settings provided optimum conditions for a larger number of organic acids compared to other settings. As it can be seen in Table 1, this was optimum for 8 acids, and 12 other acids had at least >80% response signal with these source parameters compared to their optimum settings (data not shown). For the remaining 4 acids, the responses were still in the range of 72 to 77%. The negative ion mode resulted in a more intense signal compared to the positive mode, except for acetic and propionic acid (both omitted for the purpose of this study). The internal mass reference ions used during the analysis resulted in a stable mass axis calibration, enabling the measured ions to be kept within the 2 ppm mass accuracy specified by the instrument manufacturer. Figure 1 shows the extracted ion chromatograms (EICs) of a standard mixture of the 24 organic acids analyzed and their theoretical mass-to-charge ratio used for ion extraction. The organic acids selected were chosen based on previous and our own findings in hydrolysate [10,11]. These organic acids were eluted within a narrow retention time window in a comparably short time (<12 min), as observed previously [33]. Good peak separation was achieved based on the combination of chromatographic retention time and accurate mass differences. Exceptions were the two pairs of isobaric compounds glucuronic/galacturonic acid and methylmalonic/succinic acid, which could only be distinguished by their retention time. Although no baseline separation was achieved for the pair glucuronic/galacturonic acid, the results obtained were considered satisfactory. However, the pair methylmalonic/succinic acid was almost baseline separated.

**Table 1:** **Relative signal response of the organic acids with the method source parameters chosen (gas temperature 285°C, fragmentor 75 V, and capillary 3,000 V) relative to optimum conditions determined for each organic acid**

**Compound**	**Relative signal response with method source parameters chosen compared to optimum condition [%]**
Oxalic acid	100
cis-Aconitic acid	
Maleic acid	
Glucuronic acid	
Citric acid	
Galacturonic acid	
Gluconic acid	
Pyruvic acid	
Tricarballylic acid	95
Glyoxylic acid	94
Malic acid	92
Malonic acid	91
trans-Aconitic acid	
Methylmalonic acid	
Succinic acid	90
Glycolic acid	89
Lactic acid	88
Itaconic acid	
Glutaric acid	83
Fumaric acid	81
2-Hydroxy-2-methylbutyric acid	77
Adipic acid	73
Levulinic acid	
2-Furoic acid	72

### Calibration range, limit of detection, limit of quantification

Calibration curves were generated, analyzing a set of serial dilutions from a concentrated mixture containing all 24 acids. The concentrations tested ranged between 0.01 μg/mL to 100 μg/mL (200 μg/mL for levulinic acid), and every level was run five times. Table 2 shows the linear adjustments for the 24 compounds. The lower limit of quantification (LLQ) was determined as the lowest concentration for which the obtained relative standard deviation (RSD) was smaller than 10%. The upper limit of quantification (ULQ) was determined as the highest concentration level before signal saturation. The limit of detection (LOD) was calculated as the resulting concentration after using a signal-to-noise criterion of 3. For this limit, an average noise signal of five blanks on the EIC was used. Linear fittings were possible for all acids, most of them in a range wide enough to allow quantification of the hydrolysate after 1:10 dilution. For the measurement of glucuronic acid, galacturonic acid, and glyoxylic acid only, the hydrolysate sample had to be diluted 1:100 so that the analyte concentrations were within the linear range. The LLQ ranged from 0.2 μg/mL to 2.9 μg/mL and the LOD from 0.03 μg/mL to 0.7 μg/mL. Note that the presented method only uses a 2 μL injection volume, since no sample preparation or clean-up step other than filtration and dilution is performed. This reduces the amount loaded onto the column and minimizes the contamination of the ion source and mass spectrometer by matrix compounds. When normalized to the injection volume applied, the LOD values reported here were similar or even lower compared to those of other studies using ion exclusion columns with formic or acetic acid as the eluent and MS detection [12,33,38,39]. Most linear dynamic ranges comprised two orders of magnitude, and some up to three. The regression coefficients ranged from 0.9940 to 0.9998, reflecting the good linearity of the calibration. For lactic acid an accurate LOD was not determined, since the signal obtained for the lowest concentration tested (0.01 μg/mL) was higher than the 3 times noise criterion.

**Table 2:** **Calibration results for each organic acid**

**Compound**	**Equation**	**Regression coefficient**	**LLQ (μg/mL)**	**ULQ (μg/mL)**	**LOD (μg/mL)**
Oxalic acid	y =8187.5x - 7954.7	0.9988	2.87	28.71	0.50
cis-Aconitic acid	y =21093x - 5565.6	0.9985	0.31	124.00	0.06
Maleic acid	y =84067x - 16233	0.9992	0.27	81.00	0.05
Glucuronic acid	y =14419x +1094.2	0.9993	0.53	15.60	0.11
Citric acid	y =20732x +6991.4	0.9996	0.34	102.00	0.07
Galacturonic acid	y =15778x +7460.2	0.9986	0.72	36.00	0.11
Gluconic acid	y =12550x +6863.1	0.9990	0.29	58.00	0.10
Pyruvic acid	y =14707x +10512	0.9990	0.96	48.00	0.10
Tricarballylic acid	y =26542x +1315	0.9998	0.28	55.44	0.05
Glyoxylic acid	y =4295.9x +1770.2	0.9940	1.09	19.50	0.15
Malic acid	y =32279x - 2606.8	0.9992	0.35	70.00	0.04
Malonic acid	y =29560x - 17152	0.9951	0.82	54.45	0.18
trans-Aconitic acid	y =12279x +5867.4	0.9998	0.71	47.00	0.07
Methylmalonic acid	y =34174x - 869.82	0.9992	0.25	9.90	0.03
Succinic acid	y =26782x +4284.6	0.9987	0.25	24.50	0.03
Glycolic acid	y =18633x +16302	0.9970	0.78	39.11	0.04
Lactic acid	y =23633x +25347	0.9980	0.34	34.17	<0.01
Itaconic acid	y =33879x - 2761.6	0.9993	0.23	22.50	0.10
Glutaric acid	y =34426x - 6184.7	0.9988	0.23	23.27	0.10
Fumaric acid	y =31579x +19428	0.9991	0.49	98.00	0.05
2-Hydroxy-2-methylbutyric acid	y =79616x +936.42	0.9998	0.26	50.96	0.03
Adipic acid	y =29753x - 3138.7	0.9996	0.23	46.00	0.05
Levulinic acid	y =4245.6x +11213	0.9976	1.52	152.00	0.30
2-Furoic acid	y =4437.7x +2071.3	0.9990	2.25	45.00	0.70

Lower limit of quantification (LLQ) was determined as the concentration level with a relative standard deviation (RSD) <10% (n =3). Upper limit of quantification (ULQ) was determined as the upper concentration level at which the calibration started to deviate from a linear response. Limit of detection (LOD) was determined as the resulting concentration for a signal-to-noise (S/N) =3 criterion.

For some acids, linearity was achieved only in a small range. That was the case for oxalic acid (2.9 to 28.7 μg/mL), glyoxylic acid (1.1 to 19.5 μg/mL), and 2-furoic acid (2.3 to 45 μg/ml) acid. A wider calibration range can be achieved by applying a quadratic calibration equation (data not shown and not further pursued for the purpose of this study). This is an accepted strategy as long as sufficient calibration points are used throughout the measurement range [40].

### Analytical performance characteristics and method application

The evaluation of the method was performed using a dilute acid pretreated biomass hydrolysate containing a complex mixture of innumerable compounds [8,9]. Co-eluting compounds can potentially cause signal suppression or enhancement [41] and influence the detection and quantification of the organic acids. It is known that dilute acid hydrolysate in general is rich, for example, in monosaccharides and their degradation products as well as acetic acid; these compounds can exceed the concentrations of the other organic acids by a factor of up to 1,000. Their concentrations in the present hydrolysate were 51 mg/mL xylose, 23 mg/mL glucose, 5.8 mg/mL arabinose, 0.9 mg/mL 5-hydroxymethylfurfural (5-HMF), 2.2 mg/mL furfural, and 9.8 mg/mL acetic acid. Whereas the monosaccharides (3.2 to 4.4 min), acetic acid (5.7 min), and 5-HMF (11.5 min) eluted within the 12 min suggested run time, furfural (16.7 min) and potentially other compounds eluted later. Since the method uses isocratic elution applying only one solvent and does not involve any column cleaning steps, later eluting compounds will elute during the next (or later) injection. This is an important fact to consider, because analysis is not only performed on one sample alone but rather on a set of samples that are injected sequentially. Therefore, it was more appropriate to perform a method of evaluation comprising repeatability and recovery/precision using a real hydrolysate matrix. The absolute percentage difference of the values obtained from analyzing the hydrolysate by using a 20 min isocratic LC method (ensuring furfural elution before the next injection) varied from -2.6% to 4.3% compared to the 12 min isocratic LC method (data not shown). Therefore, the longer run time did not improve the accuracy of the results or imply that later eluting compounds did not interfere with the organic acid quantification in the following run when using only a 12-min run time.

Table 3 shows the recovery results after spiking of 1:10 diluted hydrolysate with 1, 5 and 10 ppm (μg/mL) of organic acid standards. For glucuronic acid, galacturonic acid, and glyoxylic acid, a 1:100 dilution had to be applied in order to measure within the linear range. Most recoveries obtained were in the range of 70 to 130% of the theoretical yield. In this respect, the method was comparable to a previously reported method analyzing organic acids in hydrolysate using two internal standards (one deuterated, one unlabeled) with 70 to 130% recoveries after a sample clean-up step, where organic acids were extracted first by methyl tertiary butyl ether [10]. In the current study, higher recovery deviations were observed for the 1 ppm level of glyoxylic acid (426%), the 5 ppm levels of malonic acid (217%) and glyoxylic acid (324%), and the 10 ppm levels of glyoxylic acid (235%), malic acid (178%), and malonic acid (249%). In a study measuring the organic acids from plant tissues involving two ^13^C-labeled standards and time-of-flight MS detection, recoveries for three of ten organic acids (oxalic, 2-oxoglutaric, ascorbic) were reported as 39%, 44% and 22%, respectively, depending on the matrix, although other recoveries were in the range of 92 to 100% [33]. In another study applying MS/MS detection for the analysis of organic acids in plant tissue and exudates, the recoveries were in the range of 74 to 115%. However, higher deviations of 43% and 125% were observed in some samples for cis-aconitic acid and oxalic acid, respectively [39]. The application of internal standards is therefore not a guarantee for accurate recoveries. This is reasonable, since matrix effects that influence ionization can usually only be accurately compensated when an isotopically labeled standard for each analyte is used or when recoveries are determined by spike-in experiments. In the current study, the obtained absolute values, especially for glyoxylic acid and also malic acid and malonic acid, have to be interpreted carefully, although the observed recoveries have been reproduced many times. A higher analyte signal compared to the calibration sample is referred to as “ion enhancement” and is a matrix effect caused by co-eluting compounds influencing the ionization of the compound in the MS ion source. A possible cause for ion enhancement is, for example, if the standard/calibration mixture contains a larger number (or larger amount) of co-eluting compounds than the sample [41] (thus, ionization is enhanced in the “cleaner” sample matrix). In the case of hydrolysate, this was excluded, since glyoxylic acid, malic acid, and malonic acid elute in a region where the most abundant hydrolysate component (xylose) also appears. However, matrix effects are in general complex and can be attributed to more than one cause [42]. Even the instrumentation can be a reason for matrix effects; therefore, it is very possible that the same effects will not be observed on a mass spectrometer from a different vendor. Since matrix effects are common but influence the performance of the method, the evaluation of matrix effects is an important part of any analytical method involving mass spectrometry detection. Therefore, if sample clean-up steps are not performed or isotope-labeled standards are not used, spike-in experiments for recovery determination or standard addition calibration are recommended for higher accuracy of the determination of glyoxylic acid, malic acid, and malonic acid.

**Table 3:** **Recoveries obtained after spiking 1:10 diluted (1:100 for glucuronic acid, galacturonic acid and glyoxylic acid) hydrolysate with 1 ppm, 5 ppm and 10 ppm**

**Compound**	**Recovery 1 μg/mL (%)**	**Recovery 5 μg/mL (%)**	**Recovery 10 μg/mL (%)**
Oxalic acid	93.5	104.3	92.6
cis-Aconitic acid	100.0	115.9	112.3
Maleic acid	108.2	116.3	113.2
Glucuronic acid	109.8	119.4	115.8
Citric acid	89.4	104.3	95.0
Galacturonic acid	98.9	108.0	108.6
Gluconic acid	106.6	115.4	130.0
Pyruvic acid	126.4	110.7	117.9
Tricarballylic acid	94.2	111.8	120.9
Glyoxylic acid	425.6	323.9	235.1
Malic acid	108.3	117.7	178.6
Malonic acid	98.2	217.1	248.6
trans-Aconitic acid	91.6	90.6	106.3
Methylmalonic acid	98.3	105.4	109.5
Succinic acid	94.8	93.8	92.6
Glycolic acid	73.9	94.8	80.6
Lactic acid	105.4	107.4	103.7
Itaconic acid	109.9	112.8	120.9
Glutaric acid	101.3	99.9	89.3
Fumaric acid	99.2	101.6	100.0
2-Hydroxy-2-methylbutyric acid	100.2	100.9	102.8
Adipic acid	99.1	90.5	94.7
Levulinic acid	85.0	96.8	93.7
2-Furoic acid	93.5	104.3	92.6

Repeatability was determined by repeated injection of the same hydrolysate on different days. Relative standard deviations (RSDs) ranged from 0.4 to 9.2% (average 3.6%) for the intra-day experiment and 2.1 to 22.8% (average 8.9%) for the inter-day (three-day) experiment (Table 4). Overall, the averages obtained from the inter-day experiment were in good agreement with the values from the initial day (Table 4). Therefore, the method is deemed sufficiently accurate for the analysis of organic acids in hydrolysate from dilute acid pretreatment.

**Table 4:** **Average concentration of organic acids detected in dilute acid pretreated** ***Miscanthus*** **hydrolysate and relative standard deviation (RSD) of intra-day and inter-day repeatability**

	**Intra-day**		**Inter-day**	
**Compound**	**mean (μg/ mL)**	**RSD (%)**	**mean (μg/ mL)**	**RSD (%)**
Oxalic acid	48.4	2.0	47.8	4.3
cis-Aconitic acid	7.2	2.0	7.4	3.4
Maleic acid	11.4	3.1	11.3	1.2
Glucuronic acid	251.9	0.4	259.5	5.4
Citric acid	90.5	1.1	88.6	2.6
Galacturonic acid	607.5	0.7	581.8	3.9
Gluconic acid	218.0	2.7	215.5	1.6
Pyruvic acid	159.5	4.3	158.2	7.9
Tricarballylic acid	64.5	1.4	63.3	7.3
Glyoxylic acid	602.2	9.2	627.8	7.6
Malic acid	92.1	0.8	81.7	11.5
Malonic acid	27.8	1.5	26.7	14.9
trans-Aconitic acid*	98.4	1.7	103.3	6.6
Methylmalonic acid	7.6	4.4	7.6	18.7
Succinic acid	19.0	5.8	19.8	4.8
Glycolic acid	56.0	3.3	57.8	4.0
Lactic acid	31.1	4.9	38.0	7.2
Itaconic acid	5.7	5.8	6.5	13.4
Glutaric acid*	93.8	1.8	90.1	3.9
Fumaric acid*	105.8	2.5	103.6	2.2
2-Hydroxy-2-methylbutyric acid*	101.4	3.2	107.4	5.7
Adipic acid*	88.6	2.7	84.1	6.1
Levulinic acid	1114.3	1.0	1071.2	4.4
2-Furoic acid	12.2	8.8	13.1	12.9

Hydrolysate was analyzed at day 1 (intra-day, n =3) and also at three different days (inter-day, n =3). Results respresent the concentration of the non-diluted hydrolysate (“as is”). Organic acids marked with an asterisk (*) were below the limit of quantification in the hydrolysate used. For these acids, the 1:10 diluted hydrolysate was spiked with approximately 10 μg/mL standard solution.

## Conclusion

We have shown that the analysis of the profile of 24 organic acids present in dilute acid pretreated biomass hydrolysate can be achieved by a simple LC/MS method applying external calibration and minimal sample preparation comprising only filtration and dilution. Note also that the present method profiles a larger number of non-phenolic acids in the pretreatment hydrolysate than previous studies [10-12,19]. The 24 organic acids were eluted within only 12 min by isocratic elution, enabling high sample throughput. Repeatability and recovery were sufficiently accurate for most of the organic acids tested, making the method suitable for the fast determination of organic acids in hydrolysate. We envision that this method can be further expanded to a larger number of organic acids including phenolic acids, such as *p*-coumaric acid and ferulic acid, and other molecules depending on the researchers’ needs.

## Methods and materials

### Chemicals

LC/MS grade formic acid and water were obtained from Fisher Scientific (Pittsburgh, PA). Organic acids, all 99 +%, were purchased from Sigma-Aldrich (St. Louis, MO).

Hydrolysate was obtained from the National Renewable Energy Laboratory (NREL). Pretreatment conditions were: *Miscanthus* (around 1 inch size) was incubated with 1.5% (w/w) sulfuric acid at a 25% biomass loading (w/w) at 190°C for approximately 1 min, then the pressure was rapidly released. The liquid phase after filtration is referred to as “hydrolysate”.

### Liquid chromatography/mass spectrometry

Compounds were analyzed using a 1200 Series liquid chromatography system (Agilent Technologies, Santa Clara, CA) coupled to a 6520 Accurate-Mass Q-TOF mass spectrometer (Agilent Technologies, Santa Clara, CA) equipped with a dual-spray electrospray ionization source. 2 μL aliquots of the diluted samples were injected onto a Phenomenex (Torrance, CA) Rezex™ ROA-Organic Acid H + (8%) (150 mm × 4.6 mm) column equipped with a Phenomenex (Torrance, CA) Carbo-H^+^ (4 mm × 3 mm) guard column. The compounds were eluted at 55°C with an isocratic flow rate of 0.3 mL/min of 0.5% (v/v) formic acid in water (132.5 mM formic acid in water). The negative ion mode mass spectrometry conditions were: gas temperature =285°C, fragmentor =75 V and capillary =3,000 V, scan range m/z 50 to 1100, 1 scan/s. Internal mass reference ions m/z 112.9856 and m/z 1033.9881 were used to keep the mass axis calibration stable during the analysis.

### Sample preparation and analysis

A calibration mixture containing all 24 organic acids studied was prepared in 0.5% formic acid in water at approximately 100 μg/mL of each acid (200 μg/mL for levulinic acid). To determine the linear calibration range, limit of quantification and limit of detection, the calibration solution was serially diluted to 0.01 μg/mL and each concentration level was analyzed five times. The hydrolysate sample was filtered, and 100 μL were diluted with 900 μL 0.5% formic acid in water (100 μL of this dilution were further diluted with 900 μL 0.5% formic acid in water for the determination of glucuronic, galacturonic, and glyoxylic acid). The sample was then analyzed three times with and without spiking of a known standard mixture concentration and run for 12 min in order to determine analyte recovery in the presence of matrix compounds (signal suppression or enhancement). The recovery of the standard spike was calculated as ([measured amount of analyte in spiked hydrolysate] - [measured amount of analyte in unspiked hydrolysate])/[amount of analyte spiked in] × 100%.

For intra-day/inter-day comparison of repeatability, the hydrolysate sample was analyzed three times each on day one and additionally on three different days afterwards. Since trans-aconitic acid, glutaric acid, fumaric acid, 2-hydroxy-2-methylbutyric aid, and adipic acid were below the limit of quantification, the hydrolysate was spiked with about 10 ppm of these compounds.

### Data processing

The extracted ion chromatograms for the individual mass-to-charge ratios were integrated using MassHunter Quantitative Analysis software version B.05.00 (Agilent Technologies). Gaussian peak smoothing was applied with a smoothing function width of 15 and a Gaussian smoothing width of 5.

## Abbreviations

EIC: extracted ion chromatogram
LC: liquid chromatography
LC/MS: liquid chromatography coupled to mass spectrometry
LLQ: lower limit of quantification
LOD: limit of detection
MS: mass spectrometry
MS/MS: tandem mass spectrometry
QTOF: quadrupole time-of-flight
RI: refractive index
RSD: relative standard deviation
ULQ: upper limit of quantification
UV: ultraviolet
